# Computer-Aided Analysis of West Sub-Saharan Africa Snakes Venom towards the Design of Epitope-Based Poly-Specific Antivenoms

**DOI:** 10.3390/toxins14060418

**Published:** 2022-06-18

**Authors:** Albert Ros-Lucas, Pascal Bigey, Jean-Philippe Chippaux, Joaquim Gascón, Julio Alonso-Padilla

**Affiliations:** 1 Barcelona Institute for Global Health (ISGlobal), Hospital Clínic—University of Barcelona, 08036 Barcelona, Spain; quim.gascon@isglobal.org; 2 Université Paris Cité, CNRS, INSERM, UTCBS, F-75006 Paris, France; pascal.bigey@parisdescartes.fr; 3 Chimie ParisTech, PSL University, F-75005 Paris, France; 4 MERIT, Institut de Recherche pour le Développement, Université de Paris, F-75006 Paris, France; jean-philippe.chippaux@ird.fr; 5 CIBERINFEC, ISCIII—CIBER de Enfermedades Infecciosas, Instituto de Salud Carlos III, 28029 Madrid, Spain

**Keywords:** antivenom, snake bites, snake venoms, snakes, B-cell epitopes, Sub-Saharan Africa

## Abstract

Snakebite envenomation is a neglected tropical disease that causes over 100,000 deaths each year. The only effective treatment consists of antivenoms derived from animal sera, but these have been deemed with highly variable potency and are usually inaccessible and too costly for victims. The production of antivenoms by venom-independent techniques, such as the immunization with multi-epitope constructs, could circumvent those drawbacks. Herein, we present a knowledge-based pipeline to prioritize potential epitopes of therapeutic relevance from toxins of medically important snakes in West Sub-Saharan Africa. It is mainly based on sequence conservation and protein structural features. The ultimately selected 41 epitopes originate from 11 out of 16 snake species considered of highest medical importance in the region and 3 out of 10 of those considered as secondary medical importance. *Echis ocellatus*, responsible for the highest casualties in the area, would be covered by 12 different epitopes. Remarkably, this pipeline is versatile and customizable for the analysis of snake venom sequences from any other region of the world.

## 1. Introduction

Snakebite envenomation is a deadly condition categorized as a neglected tropical disease by the WHO since 2017 [1,2]. It disproportionately affects tropical countries in Africa, Asia, Central and South America, and Oceania, yearly causing over 100,000 deaths and permanently disabling ~400,000 people [3]. At present, the only effective therapeutic treatment are antivenoms derived from immunized animal sera, a technique developed more than a century ago [4]. However, a decades-long widespread antivenom crisis prevents effective treatment for snakebite victims in poorly developed countries in terms of both availability and quality of the products [5,6]. An important factor in this crisis is that the current manufacturing process entails recurrent mild inoculations of large animals with venom extracted from snakes kept in captivity. This involves high production and distribution costs, in large part because the manufacturing processes and premises are often unavailable in the regions in most need of the antivenoms [6].

As a result of predator–prey coevolution dynamics, snake venoms are cocktails of peptides and proteins from different protein families, with each snake species having different proportions of each protein family [7]. Even within the same species, venom composition varies in geographically unrelated animals [8,9]. Currently, the WHO guidelines for the production, control and regulation of snake antivenom immunoglobulins recommend the mixing of venom from different origins, ages and sexes to compensate for this (World Health Organization, 2017). However, usually, it is just a few of the proteins in the cocktail that are responsible for the majority of the envenomation symptoms, but antibodies in antivenoms are generated against the whole range of molecules in it [10]. Venom-dependent antivenom production does not compensate for this, which might result in less effective products and higher dosages needed, with the associated higher risks of adverse effects and increased treatment costs [6,11]. To bypass these shortcomings, several venom-independent approaches are being evaluated [12], although no commercialized product has yet been approved based on them. One of the most promising strategies stands on the use of genetic immunization with constructs encoding the specific venom toxins of interest [12]. Instead of using peptides or full-length recombinant proteins, which synthesis or expression is highly costly, synthesis of DNA is comparably cheaper. Interestingly, previous studies have demonstrated that sera produced upon plasmid DNA (pDNA) immunization can neutralize snake venom deleterious effects, either using whole toxin cDNA as immunogen [10,13,14,15] or genetic constructs carrying multiple epitopes from specific toxins [16,17].

Remarkably, multi-epitope genetic constructs may contain dozens of epitopes from multiple origins, which upon delivery would be able to elicit a targeted response against them. Thanks to the availability of several bioinformatics tools, the selection of those epitopes can be undertaken computationally at a low cost, for instance, guided by sequence conservation criteria and protein toxicity features. Indeed, a multi-epitope construct that takes into account these requirements could, in theory, neutralize the venom from any given species, independently of their geographical origin. Not only that, considering that cross-neutralization has been observed in previous experiments with pDNA immunization [13,15,17], if the correct selection of epitopes is achieved, a single construct would have the potential to provide coverage against envenomation by several snake species.

In this study, we present a flexible and versatile computational pipeline that relies on publicly available snake venom protein sequences and bioinformatics resources for the analysis and selection of a series of peptides of interest to be carried on by a pDNA construct. Such selection relies on a knowledge-based strategy encompassing evolutionary conservation, protein annotation and lack of sequence identity with susceptible preclinical evaluation models or antivenom-producing hosts. Furthermore, for the prediction of B-cell epitopes, we concede major relevance to the availability of 3D structures or high-quality models to follow a structure-based approach [18]. We use venom proteins from West Sub-Saharan African medically important snakes as input for the pipeline as the first step towards the hypothetical development of a novel epitope-based antivenom product. For this, we ultimately provide a curated list of 41 B-cell epitopes found in 42 different proteins from 14 distinct snake species, which, encompassed in a pDNA construct, could give rise to protective antibodies against snake venom toxins from the studied region leading to a poly-specific antivenom therapy.

## 2. Results

**Figure 1:**
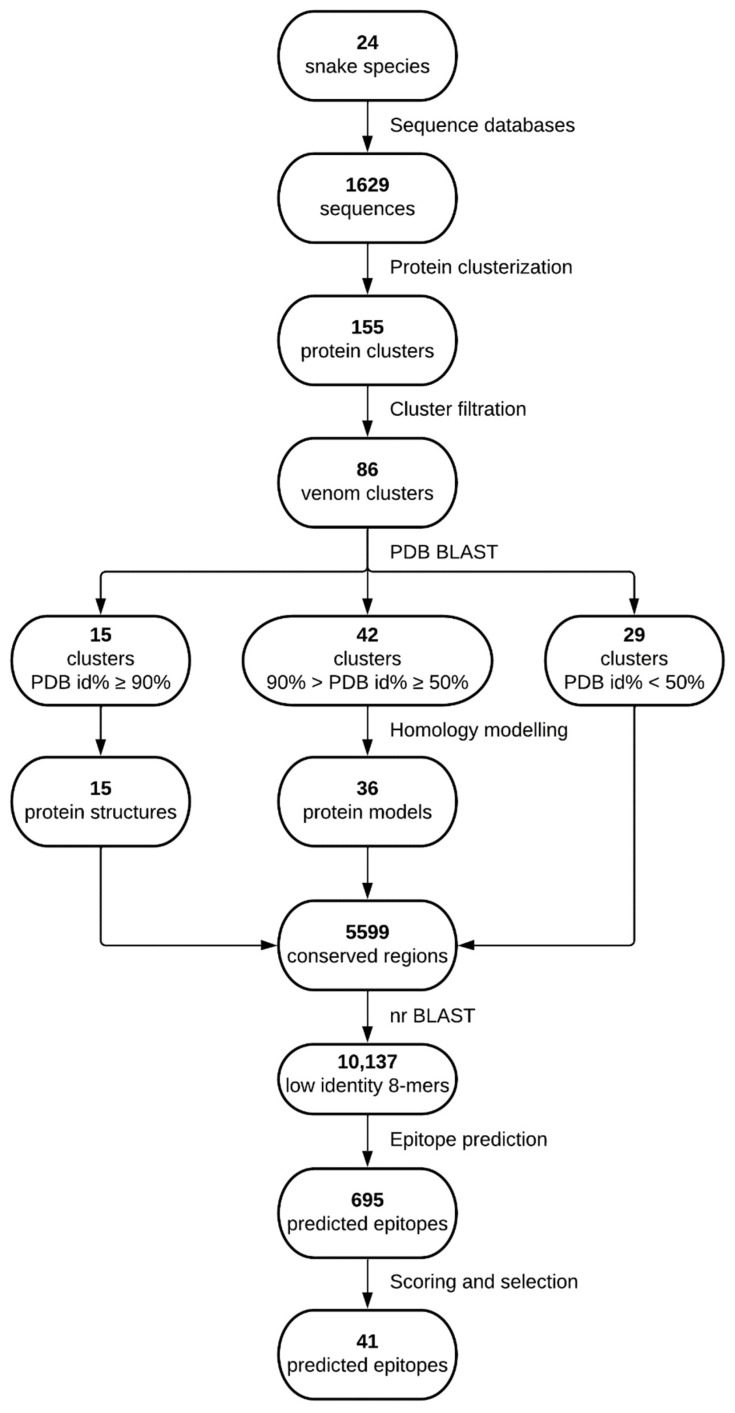
Study flowchart summarizing the procedures followed to reach the selected epitopes. Lucidchart was used to make this figure.

**Figure 2:**
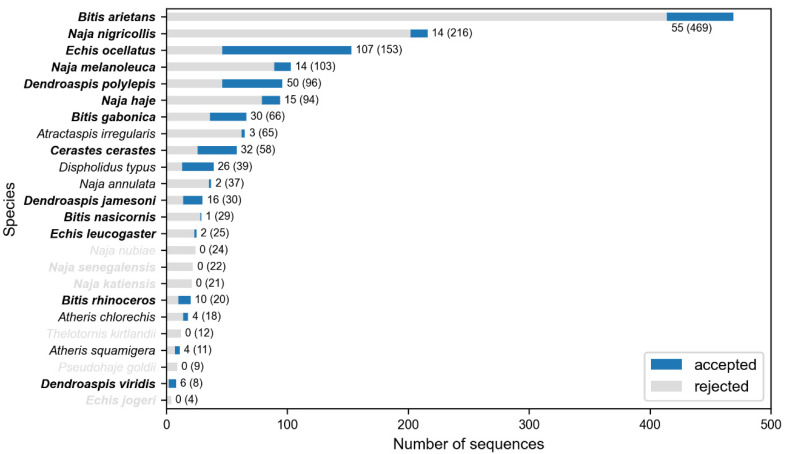
**Protein sequences available for each of the snake species studied.** The proportion of venom sequences is shown in blue, while the proportion of non-venom discarded sequences is shown in gray. Numbers near each bar indicate the accepted sequences per species over the total available protein sequences for that particular snake (number in parenthesis). In the Y-axis, species highlighted in bold are of class I medical importance. Species names in gray indicate that there were no sequences accepted for these.

All medically relevant snake species from West Sub-Saharan Africa are summarized in Table 1, and a general overview of the followed process is presented in Figure 1. A total of 1629 snake protein sequences were downloaded, 154 from UniProt [19] and the other 1475 from NCBI [20], 41 of which originated from the Protein Data Bank (PDB) [21]. Sequences were unequally distributed among the studied species, with the top three species (*Bitis arietans*, *Naja nigricollis* and *Echis ocellatus*) providing more than 50% of the sequences (Figure 2). Sequence clustering generated 155 protein clusters, which, once filtered to only keep venom-containing sequences, left 86 of them. This drastically reduced the number of available protein sequences in some species and even left out others from further analysis, which is the case of *N. nubiae*, *N. senegalensis*, *N. katiensis*, *T. kirtlandii*, *P. goldii* and *E. jogeri* (Figure 2). The mean depth of the clusters was 4.55 sequences (median 3 sequences) per cluster, the minimum depth being 1 sequence and the maximum 22.

The outcome of a BLASTP [22] search against the PDB database using the retrieved reference sequences from each cluster yielded 15 clusters with a PDB entry identity above or equal to 90%; 42 with an identity between 90% and 50%; and 29 with identity below 50% (Supplementary Table S1). Those 42 reference sequences with identities between 90% and 50% were used to generate models by means of homology modeling using their corresponding PDB template. As a result, 36 3D models with different degrees of reliability were generated (Supplementary Table S1). The 86 venom clusters were split into 5599 totally conserved regions, from which 16,460 8-mers, the minimum length we considered for the B-cell epitope predictions, were generated and blasted against the organisms described in the Methods Section 4.5. This resulted in 10,137 8-mers with an identity < 75%, a maximum of six out of eight identical positions, to any protein sequences from those organisms. 

**Figure 3:**
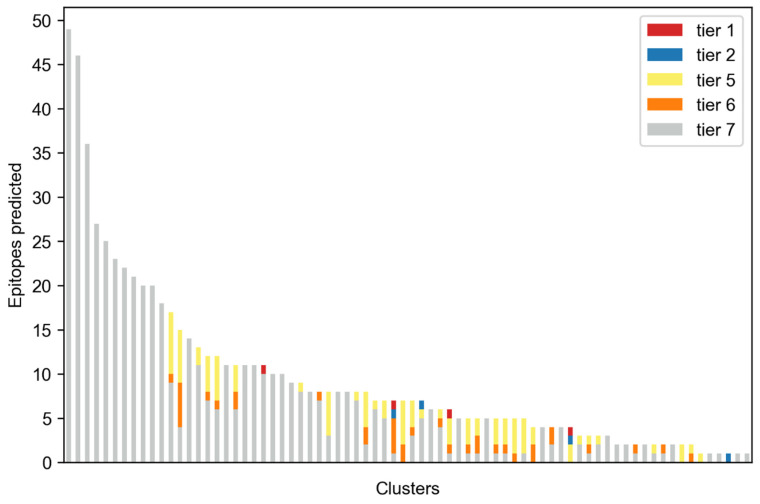
**Total epitopes predicted.** Each column represents a different cluster. Colors indicate the highest tier of the predicted epitopes.

Using features from both structure and sequence information, while keeping in mind conservation as well as low identity with selected organisms, we predicted 695 epitopes in the conserved fragments. Only 74 of the 86 clusters produced at least one epitope. These were classified according to their characterization into seven tiers as described in Methods Section 4.5. Looking at the best tier for each predicted epitope, it can be observed that most were predicted by the sequence-only method (tier 7), and no epitopes with tiers 3 or 4 were predicted at all (Figure 3).

**Figure 4:**
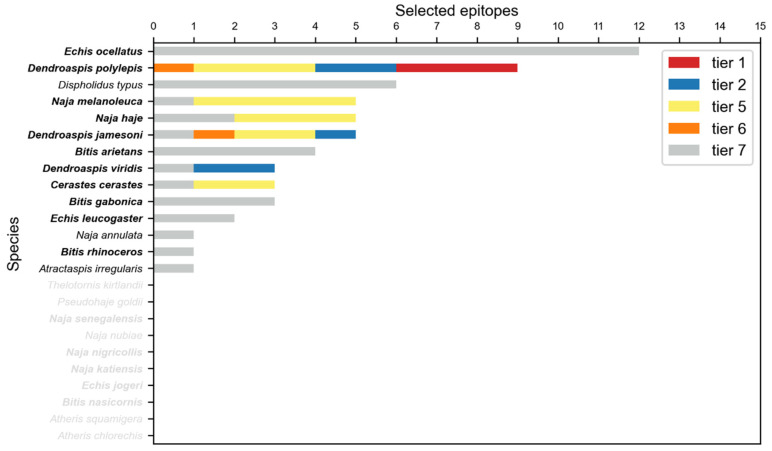
**Snake species covered by the selected epitopes.** Color legend depicts the highest tier of the predicted epitopes that cover a particular species. Note that any given epitope might cover different snake species. Species without accepted clusters are not shown.

**Figure 5:**
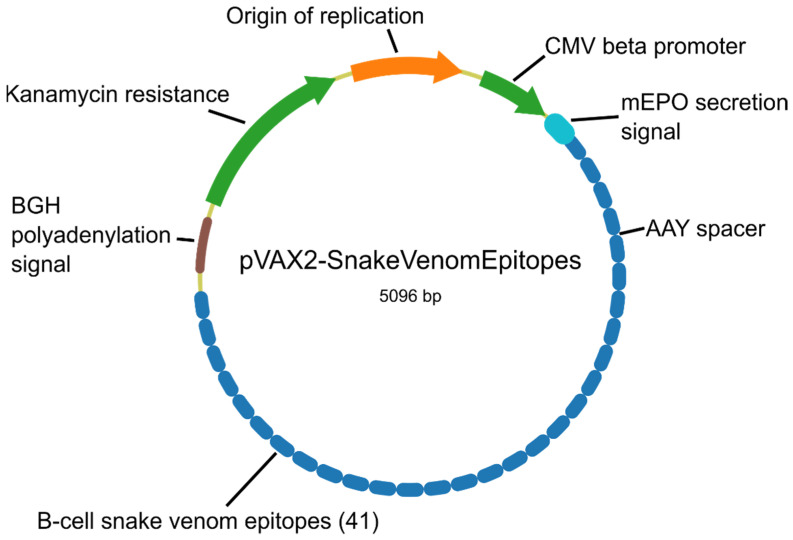
Map of the proposed genetic construct to deliver the selected epitopes.

Epitopes were merged and then scored using their protein and species origin, length and substring identity with IEDB validated epitopes. The best epitope per cluster was selected, and only clusters from relevant toxin families were considered, as described in Methods. The final selection contained 41 peptide sequences (Supplementary Table S2), which covered 42 out of 86 venom clusters. The selection also covered 53.9% of the snake species categorized by the WHO, 11 out of 16 of those considered of highest medical importance and 3 out of 10 of those considered as class II (Figure 4). In particular, *Echis ocellatus*, responsible for the largest casualties in West Sub-Saharan Africa, is covered by 12 epitopes. All epitopes were mapped to their corresponding structures and visualized using PyMol [23] (Supplementary Figures S1 and S2). A DNA construct encompassing all selected epitopes was designed as described in Methods (Figure 5, Supplementary File S1).

## 3. Discussion

Snakebite envenoming is currently treated with antivenoms obtained from sera of large animals inoculated with venoms, which must be milked from the corresponding snakes [3,4]. Antivenom therapy is often too expensive for health systems or for the snakebite victims most affected by this neglected disease [6]. Moreover, there is a trade-off between antivenom potency and its price. Usually, cheaper products are less potent, thus translating into higher dosages needed or even products that are completely ineffective [6]. In addition, the poor quality of some antivenoms has also been evidenced by the appearance of life-threatening adverse reactions upon administration, which very worryingly undermines the trust of healthcare workers in snakebite medical treatment [6,11,24]. Thus, it is fundamental to maintain high standards of production and quality control while trying to keep prices as low as possible.

Herein, we present a versatile in silico pipeline from which to eventually achieve cost-effective, toxin-targeted, potent, epitope-based antivenoms that would be obtained in a venom-independent manner. Using a genetic construct to immunize the animals would save the need for a herpetarium, sidestepping the periodical and risky procedure of extracting the venoms, precluding the danger to handlers and likely reducing costs. Additionally, taking into account that individual variability can affect venom composition [25,26], antivenom production based on synthetic methods would contribute to the homogeneity of batches. In terms of costs, using pDNA would also be advantageous, considering it is much cheaper to synthesize than peptides or recombinant proteins to immunize the animals [12]. A major feature of adequately treating snakebite victims is the identification of the snake responsible, but this is usually not possible. Therefore, the use of polyvalent antivenoms is of utmost importance [27]. Such antivenoms should be raised against all snake species of medical importance within a region or country [28]. For this, the diversity in toxins from distinct protein families and from different snake species must be taken into consideration. There stands the rationale of identifying evolutionarily conserved sequences among all available venom protein toxins from a group of snake species of medical importance at the regional/national level. Moreover, a polyvalent antivenom strategy should as well contribute to saving costs due to the development and production of fewer products that can be functional against numerous snakes.

An issue with some current antivenoms is lack of potency, mainly due to the fact that the immune system of the venom-inoculated animal generates a response against the whole venom protein cocktail. Ultimately, this “decoyed” potency entails that more antivenom vials must be administered to treat envenomation resulting in the occurrence of intolerance reactions or even anaphylactic shocks [29], and the use of more vials increases the cost of treatment. Additionally, within the venom cocktail, there are high molecular weight proteins that are more immunogenic by nature and would elicit a biased response towards them. This means that, for species in which highly immunogenic proteins are not the main source of toxicity, there will be a large number of ineffective immunoglobulins in the final product [12,30,31,32,33]. This phenomenon is particularly relevant when developing poly-specific antivenoms, given that some species might eclipse others, as it occurs with vipers over elapid species. Neurotoxins of the latter have comparatively low molecular weights versus vipers’ cyto- and hemotoxins [34]. An epitope-based approach could circumvent these issues by focusing on the immune response towards specific epitopes of relevant venom proteins.

From this perspective, we scored and selected the best potential epitopes of as many as possible snakes of medical interest from West Sub-Saharan Africa, taking into consideration the most relevant toxin protein families. Interestingly, our clustering and conservation approach shows that some of the predicted epitopes are conserved in proteins from the same family inside a given species and even in proteins from different species. Even though this is somewhat expected at the genus level (e.g., *Dendroaspis* spp.), we also found epitopes that would cover proteins from different genera, such as metalloproteinases from *Bitis arietans* and *Echis ocellatus*. This would allow covering several toxins using a low number of sequences. Additionally, with this methodology, it would be possible to generate antibodies against protein families absent in current antivenoms, such as in the case of sarafotoxins in *Atractaspis* spp., either due to difficulties milking the venom or because the toxins show low immunogenicity. While experimental validation is pending, the peptide list presented here, the result of a bioinformatics analytical pipeline, is the first step in the development of innovative epitope-based polyvalent snake antivenom for West Sub-Saharan Africa.

Like any other data analysis work, a limitation of the study is the inherent bias of snake sequences in the repositories. This is especially true regarding African snakes, where venom sequence availability is scarcer than for snakes from other continents. Resources such as the UniProt Animal Toxin Annotation Project can provide a high level of detail on venom protein composition, but our analysis would certainly benefit from larger proteomic databases of snake venom sequences. Anyhow, luckily (and expectedly), the snakes with a higher number of sequences in the databases were *Echis ocellatus* and *Bitis arietans*, which have been estimated to be responsible for most of the casualties in Africa [3,35].

Remarkably, the substring IEDB legacy-experimentation approach found three matches with already validated B-cell epitopes (VHQCNCGA and GMNHDGNQCNCG from *E. ocellatus* and MAHDGNQCNCG from *B. gabonica*). A closer inspection of other predicted epitopes revealed that some short motifs within them are indeed part of other already validated epitopes. For instance, it is interesting to find that motif ATCP, present in the predicted epitopes ATCPKPTNVRETI and AATCPKVK that cover *Dendroaspis* species and which have been predicted through 3D features, has been found in validated 3FTXs epitopes from the Asian *Bungarus multicinctus* and the American *Micrurus corallinus* [36,37]. Nonetheless, a limitation of the study is that most of the epitope sequences identified were predicted and yet await experimental validation of their biological function. While the prediction of B-cell epitopes may not be as robust as that of T-cell epitopes (particularly CD8^+^ T cell epitopes), our pipeline involves multiple parameters and a scoring system to promote the best possible sequences. In this respect, some of the most important aspects of B-cell epitopes are their accessibility to the protein surface and their flexibility [18,38], and taking both into consideration would aid in better predictions. These parameters are calculated through 3D protein structures, but only 15 protein clusters showed high identity with PDB structures. Fortunately, about half of the clusters’ reference sequences blasted with an identity between 50% and 90% to PDB hits, and for those, we used structure homology modeling. These thresholds are a trade-off between disposing of protein structures and sequence accuracy. A threshold of 90% identity ensures that differences between a reference sequence and its PDB hit can be reduced to a minimum, and in most cases, these differences are given by one or two residues, usually in the termini. On the other hand, a 50% low-end cutoff is given because models with lower identities had increasing lower quality using homology modeling. Each sequence was modeled five times, and their loops were refined four extra times, generating a total of twenty models per sequence, from which we kept the best one. Residue flexibility can be approximated using a PDB structure B-factors [18,39], but these are lost in the modeling process. Accessibility, however, can still be calculated from accurate models by computing the relative solvent accessibility. For the tier classification of epitope sequences, these structural features were accompanied by hydrophobicity and epitope propensity estimations, aimed at classifying the best peptides on a consensus methodology. Yet it must be noted that it was not possible to model some of the sequences, especially in the case of large metalloproteinases. For these, prediction of epitopes solely relied on amino acid sequences and thus received a poorer scoring for prioritization. This caused that selected peptides for some vipers, specifically for the highly relevant *E. ocellatus*, have a higher proportion of lower quality (tier 7) epitopes. In future studies, recent advances in 3D protein modeling such as AlphaFold2 might aid in the structure-based prediction of epitopes from those large proteins [40]. All selected epitopes predicted from 3D structures were mapped onto their corresponding structure and visualized using PyMol (Supplementary Figures S1 and S2). While most appear to be clearly accessible, there are examples in which a small part of the epitope sequence is buried, a consequence of using the mean relative solvent accessibility in the epitope predictions.

Notably, the prioritization process included a step to deselect those sequences with high identity to proteins from humans and animal hosts commonly used in antivenom development or production. The purpose of this is two-fold: first, lower identity between foreign and host proteins is desired to decrease the chance of cross-reactivity against hosts’ own proteins [41,42]. Secondly, dissimilar sequences are more likely to be immunogenic due to not being subjected to immune tolerance, as illustrated by the fact that lower immune responses have been reported against bacteria from the human microbiome [43]. Ultimately, the approach served to construct an “ideal” venom immunogen, composed of the main antigens of relevant toxin families covering many dangerous species of this region, which no “natural” venom can do. The next step would be to validate these epitopes in vivo, and for this purpose, we designed a preliminary DNA construct that encompasses these sequences and that has been optimized for mice codon usage. Upon validation, a refined epitope construct can then be designed, and further computational analyses could be carried out, such as molecular dynamics and peptide-protein interaction simulations [44]. Importantly, the pipeline was designed in a modular fashion so that it is flexible and versatile and can be swiftly adapted to any region, number or type of venomous species. This same procedure could be applied to study snake venoms from India, one of the most afflicted countries by snakebite mortality in the world [45].

## 4. Methods

### 4.1. Collection of Protein Sequences from West Sub-Saharan Snakes

West Sub-Saharan Africa region, as established by the WHO, consists of Benin, Burkina Faso, Cameroon, Côte d’Ivoire, The Gambia, Ghana, Guinea, Guinea-Bissau, Liberia, Mali, Mauritania, The Niger, Nigeria, Sao Tome and Principe, Senegal, Sierra Leone, and Togo. First of all, we obtained the list of snake species of medical importance from that region with geographically driven searches in the WHO Snakebite database [last consulted on 6 October 2021] [46]. Snake species were categorized according to their medical importance as highest (1) or secondary (2). In order to download the corresponding protein sequences, the taxonomy identifier (taxid) for each species was retrieved. All species are summarized in Table 1. Some of the species did not have any associated taxid, probably due to taxonomical incongruences.

Protein sequences from identified species were retrieved from the UniProtKB/Swiss-Prot Animal Toxin Annotation Project, a highly curated database that contains venom and toxin protein sequences from many snake species, among other animals [47]. Each species’ sequences were downloaded using the following query: “(keyword:toxin OR annotation: (type:“tissue specificity” venom)) taxonomy: “{taxid}” AND reviewed: yes”, where {taxid} is replaced by each taxid. The query was launched programmatically so that the returned results were formatted as FASTA. All sequences downloaded from this database were labeled as such. Additionally, the NCBI protein database [20] was queried to obtain all protein sequences from a specific taxid, independently from their level of annotation. The Entrez utilities [48] were used to access the database with the following query: “txid{taxid}[Organism:exp]”, where {taxid} is again replaced by each taxid. Sequences downloaded from NCBI contain entries from different databases, including UniProt, and as such, any repeated entries were discarded. Special attention was paid to the sequences that came from the Protein Data Bank (PDB) [21], as they have an associated 3D structure and were labeled correspondingly.

### 4.2. Clustering, Filtering and Multiple Sequence Alignments (MSAs)

In order to reduce redundancy and analyze sequence conservation, all sequences were clustered using CD-HIT [49]. Hierarchical clustering was performed from 0.95 to 0.70 identity cutoffs with 0.05 increments. Word size was set to 2 (option -n 2), length cutoff to 0 (-s 0), and each sequence was grouped into the most similar cluster that met the threshold (-g 1). Each cluster was then assessed to discern whether its members originated from venom proteins or not. Any cluster that had at least one member from the UniProt Animal Toxin Annotation Project passed automatically, including clusters with just one member. Otherwise, two criteria were used to accept or discard clusters. First, any cluster with only one member was automatically discarded. For the second criterion, sequence descriptions from cluster members were retrieved from NCBI. A keyword list with commonly found words in venom proteins was composed, consisting of 3ftx, c-type lectin, crisp, cysteine-rich, disintegrin, hyaluronidase, jerdostatin, kunitz, metalloprotease, metalloproteinase, oxidase, phospholipase, pla, protease, snaclec, svmp, toxin, vascular endothelial growth factor, vegf and venom. Another list with common words in proteins that were not from venom was also composed: cmos, cytochrome, homeobox, nadh, neurotrophin, oocyte, opsin, periplakin, prolactin, recombination and ubinuclein. The latter keywords were frequently found in protein sequences of basic biological machinery that originated from phylogenetic studies, and thus they were not suitable for this study. Any protein description that had at least one keyword was tagged as accepted or rejected according to each list. Clusters with no rejected members and at least one accepted member were selected. Similarly, clusters with at least one rejected member and no accepted members were discarded. Notably, no cases were reported where both accepted and rejected proteins were found within the same cluster.

All finally selected venom clusters were aligned using MUSCLE [50] with standard settings and formatted as aligned FASTA files (Supplementary File S2). Some sequences contained unknown residues, marked with “X”s, so any alignment position that only contained “X” or “-”, hence causing an artificial gap, was deleted. A consensus sequence for each alignment was obtained from the most common residue in each position. Every sequence from the alignment was then scored by pairwise alignment with the consensus, giving 1 point for each identical residue. That sequence with the highest score was selected as the reference sequence for that cluster. In the case of a draw, priority was given to sequences from PDB, the UniProt Animal Toxin Annotation Project, and the rest of the NCBI sequences, in that order.

### 4.3. Conservation Analysis

Each aligned cluster was processed to analyze sequence conservation with the Shannon entropy parameter (*H*) [51,52]. Each sequence was analyzed to set the actual alignment start and end, discarding all “-” positions at both N- and C-terminal ends that appeared due to short sequences. For each position, *H* was calculated only by taking into account residues that did not correspond to terminal gaps. While any real gap was taken into account in the entropy calculation, unknown residues (marked with “X”s) were not considered (Supplementary Figure S3). The entropy value for each position was stored for later use.

### 4.4. PDB BLASTP and Modeling

A BLASTP [22] web search against the PDB database was launched programmatically using the reference sequences from each cluster under default parameters (BLOSUM62 matrix, e-value of 10, word size of 3, gap open 11 and gap extend 1, with composition-based score adjustment), and limited to returning only hits from the clade Serpentes using the query: “txid8570[ORGN]”. The best 3D structure hit for each sequence was chosen according to the highest identity percentage, calculated as the number of identities of a hit divided by the total length of the queried sequence. Each reference sequence was then classified into three separate categories depending on the identity of the PDB hit: (i) identity higher or equal to 90%; (ii) identity between 90% and 50%; (iii) and identity below 50%. PDB hits with high identity (≥90%) to clusters’ reference sequences were directly used for further predictions without previous modeling, while clusters with no relevant PDB hits (identity < 50%) were only used to predict epitopes via sequence-based methods.

Protein models were generated for the clusters with a 3D structure identity that fell between 90% and 50% with the homology modeling software Modeller [53,54]. The reference sequence and the PDB sequence were aligned by the software so that overhangs from the former that were not present in the PDB were edited out, leaving only three residues on each side that were modeled ab initio. Five models were generated for each alignment, keeping only those with a GA341 score [55], which ranges from 0 (worst) to 1 (native-like), above 0.9. Each of these models underwent a loop refinement process that generated four refined models. In total, twenty models were generated for each alignment. The best-refined model for each preliminary model was selected according to the lowest DOPE-HR score [56], which has an arbitrary scale and can only be used to compare models from the same alignment. Then, the best-refined model overall was selected according to the lowest normalized DOPE scores and used for further analysis. Preliminary models that did not contain loops, as detected by Modeller, were not refined and were selected only according to the lowest DOPE-HR score.

### 4.5. B-Cell Epitopes Prediction and Features for Epitopes Characterization and Triage 

BepiPred2 [57] was run over all the reference sequences with default parameters. Hydrophobicity of each position was calculated using a normalized hydrophobicity scale [58] from 0 to 1, with a window size of 7 (precluding the first and last three residues having their hydrophobicity calculated). For the 3D structures, both those from PDB and those generated by homology modeling, the relative solvent accessibility (RSA) was calculated using NACCESS [59] under default parameters. Finally, the B-values, or temperature factors, of the PDB structures were also retrieved and standardized using the following formula:

*Z_B_* = (*B* − *μ_B_*)/*σ_B_*, (1)

in which *Z_B_* is the new standardized *B*-value, *B* is the raw *B*-value, and *μ_B_* and *σ_B_* are the mean and standard deviation of all the *B*-values of the structure, respectively. These were used as a measure of flexibility [18,39]. Since all these features were calculated from raw sequences or 3D structures, the results were realigned accordingly into each cluster using MUSCLE.

In this study, all peptide sequences selected were based on absolute conservation within MSA, so we used *H* = 0 as a cutoff. Upon defining totally conserved peptide sequences of all reference sequences, these were split into conserved fragments and considered separately. A minimum length of eight residues was established for any given epitope, so shorter conserved fragments were automatically discarded. Then, all possible 8-mers from a given fragment were calculated for all conserved fragments.

To identify potential cross-reactive peptides, this 8-mer database was locally blasted against human (taxid 9606), chicken (taxid 9031), rabbit (taxid 9986), horse (taxid 9796), donkey (taxid 9793), dromedary (taxid 9838), llama (taxid 9844), mouse (taxid 10090) and sheep (taxid 9940) non-redundant sequences protein databases, in separate blast searches. The blastp-short task was used because of the short length of the sequences. In addition, these were blasted using the PAM30 matrix, a word size of 2, no composition-based statistics, and gap penalties of open 9 and extend 1. An expect value (e-value) threshold of 10,000 was used. The identity of each queried 8-mer to a given hit was calculated by dividing by eight (the queried length) the number of identical positions, minus any gaps that appeared. The hit with the lowest e-value was selected as the representative for each 8-mer. This meant that each 8-mer had seven hits associated, one for each blasted organism.

Epitopes were predicted by analyzing each conserved fragment 8-mer by 8-mer, calculating the mean value for each feature from the eight positions. For any given 8-mer, a tier value was selected according to the established thresholds of flexibility (≥1 in the standardized B-value scale), RSA (≥50), hydrophobicity (≤0.45, the normalized value for glycine), and BepiPred2 score (≥0.5) (Table 2). A new epitope started with the first 8-mer and appended new positions from the subsequent overlapping 8-mers. Since any given position could be part of several 8-mers, the best tier was kept for a specific position. This way, each position of a predicted epitope could show different levels of “quality”. Any 8-mer that did not fill any tier requirements was discarded, and the newly predicted epitope stopped at the previous 8-mer. Similarly, if it had a BLAST hit identity above 75% for any of the blasted organisms (a maximum of six positions out of eight), the 8-mer was also discarded. This was done to limit the similarity with peptides found in the blasted organisms in order to (1) lower chances of cross-reactivity and (2) improve immunogenicity [51]. Any 8-mer remaining in the conserved fragment was considered for a new epitope (Supplementary Figure S4).

### 4.6. Epitope Scoring and Selection

We queried the Immune Epitope DataBase [60] to download any positively found B-cell epitopes that originated from the Serpentes clade (taxid 8570) (last search: 19 October 2021). These were downloaded from the “assays tab” in order to keep information on specific assays. Epitopes recorded in IEDB and predicted epitopes were matched as substrings of one or the other, considering full sequences of both.

On the other hand, we classified each cluster, with its corresponding reference sequence, into one of ten venom protein families: three-finger toxins (3FTX), Cysteine-rich secretory proteins (CRISP), disintegrins (DIS), Kunitz peptides (KUN), L-amino acid oxidases (LAAO), natriuretic peptides (NP), phospholipase A2s (PLA2), snake C-type lectins (SNACLEC), snake venom metalloproteinases (SVMP) and snake venom serine proteases (SVSP). For this, the protein description and features data of each reference sequence were consulted on the NCBI database, parsed, and compared to lists of keywords corresponding to each family (Supplementary Table S3). If there was any keyword match, the protein was classified accordingly. No instances of multiple families were found, and clusters that could not be classified were labeled as “OTHER”. Additionally, a table with the venom composition for each studied species was adapted from Tasoulis and Isbister [7] (Supplementary Table S4). Mean proportions were calculated for individuals of the same species originating from different regions. Species without annotated venom compositions were assigned values according to the mean proportions of their snake family (Viperidae and Elapidae). For *Atractaspis irregularis* (Lamprophiidae), the same proportions were assigned to each protein family.

For the comparison and selection of the best epitopes, these were scored as follows:

1. For predicted epitopes in which their best tier value (Table 2) was between 1 and 4 inclusive, three points; for tiers 5 and 6, two points; and for tier 7, one point.
2. If a predicted epitope covered two species, one extra point was given. If it covered three or more, two extra points were given.
3. If the cluster depth (number of peptide sequences within a given cluster) was higher than 10, two extra points were given. If it was between 5 and 10, one extra point was given.
4. If any predicted epitope was found inside an IEDB epitope or the other way around, three extra points were given.

This baseline score, which ranged from 1 to 10, was multiplied by the corresponding proportion of each venom family in a particular snake species. If multiple species were covered, the proportion was calculated as the mean between the covered species. Then, the resulting epitope scores were normalized from 0 to 100 and rounded to the nearest integer. Finally, any epitope found inside a bigger epitope was merged, keeping the best score of the merged pair. To compose a final list of epitopes, the highest scored epitope for each venom cluster was selected. Any predicted epitope that had a match with validated epitopes from IEDB was automatically selected. Since the most toxic families are 3FTXs, SVMPs, SVSPs and PLA2s [12], only epitopes from these families were considered for the final list. In addition, epitopes that originated from Kunitz-type peptides of *Dendroaspis* spp. and sarafotoxins from *Atractaspis* spp. were also kept due to their relevance in these particular species [7]. Noteworthy, a single predicted epitope might be capable of covering several clusters due to the merging events mentioned above. In the case of a draw, longer epitopes were prioritized over shorter ones.

### 4.7. Genetic Construct and Codon Optimization

We designed a genetic construct encoding the 41 selected B-cell epitopes shaped like a “string-of-beads” [16]. AAY linkers were included to separate the epitopes as these should promote epitope presentation and reduce the formation of junctional epitopes [61]. A secretion signal from the murine erythropoietin (mEPO) was included upstream of the epitopes “string-of-beads”, as secretion is required to reach a high antibody titer after genetic immunization, and mEPO signal has proven very efficient in raising efficient antibodies against a *Plasmodium falciparum* antigen [62].

Thinking of the next experimental proof-of-concept, the full peptide sequence of this ensemble was then subjected to the Genscript GenSmart™ Codon Optimization Tool (https://www.genscript.com/tools/gensmart-codon-optimization (accessed on 6 June 2022)) for codon optimization using *Mus musculus* as the host organism. The resulting nucleic acid sequence was then inserted into a eukaryotic expression plasmid based on the InVitrogen pVAX2 backbone. This plasmid backbone is commonly used as an in vivo mammalian expression vector and is used in the first and only DNA vaccine approved for human use against the SARS-Cov-2 coronavirus, ZyCov-D [63]. Briefly, this plasmid contains upstream the coding sequence, a cytomegalovirus (CMV) promoter sequence that governs its transcription, and a Kozak sequence that functions as a protein translation initiation site and is located around the AUG start codon. At the 3’-end downstream of the coding sequence, there is a bovine growth hormone (BGH) poly-adenylation (polyA) signal to tag the growing mRNA molecule with a polyA tail.

## Figures and Tables

**Table 1:** West Sub-Saharan Africa snakes categorized by the WHO as medically important.

Species	Common Name	Taxid	Family ^1^	Cat. ^2^
*Bitis arietans*	Puff adder	8692	V	1
*Bitis gabonica*	East African Gaboon viper	8694	V	1
*Bitis nasicornis*	Rhinoceros viper	8695	V	1
*Bitis rhinoceros*	West African Gaboon viper	715877	V	1
*Cerastes cerastes*	Horned viper	8697	V	1
*Dendroaspis jamesoni*	Jameson’s mamba	8623	E	1
*Dendroaspis polylepis*	Black mamba	8624	E	1
*Dendroaspis viridis*	Western green mamba	8621	E	1
*Echis jogeri*	Joger’s carpet viper	696809	V	1
*Echis leucogaster*	White-bellied carpet viper	504457	V	1
*Echis ocellatus*	West African carpet viper	99586	V	1
*Naja haje*	Egyptian cobra	8639	E	1
*Naja katiensis*	West African brown spitting cobra	409859	E	1
*Naja melanoleuca*	Forest cobra	8643	E	1
*Naja nigricollis*	Black-necked spitting cobra	8654	E	1
*Naja senegalensis*	Senegalese cobra	862238	E	1
*Atheris broadleyi* ^ 3 ^	Broadley’s bush viper	NA	V	2
*Atheris chlorechis*	West African bush viper	110216	V	2
*Atheris squamigera*	Variable bush viper	110225	V	2
*Atractaspis irregularis*	Variable burrowing asp	512568	L	2
*Dispholidus typus*	Boomslang	46295	C	2
*Naja annulata*	Banded water cobra	8609	E	2
*Naja nubiae*	Nubian spitting cobra	186441	E	2
*Pseudohaje goldii*	Gold’s tree cobra	1545503	E	2
*Pseudohaje nigra* ^3^	Black tree cobra	NA	E	2
*Thelotornis kirtlandii*	Forest vine or twig snake	292880	C	2

^1^ Taxonomic families: V—Viperidae; E—Elapidae; L—Lamprophiidae; C—Colubridae. ^2^ Category of medical importance as stated in the WHO website. ^3^ These species did not have a taxonomy identifier and were thus excluded from this study. NA—not available.

**Table 2:** **Features considered for tier-ranking of the predicted epitopes.** The presence of an X inside a cell indicates whether that particular feature is fulfilled within the corresponding threshold in that specific tier.

Tier	BepiPred2	Hydrophobicity	RSA	Flexibility
Tier 1	X	X	X	X
Tier 2	X		X	X
Tier 3		X	X	X
Tier 4			X	X
Tier 5	X	X	X	
Tier 6	X		X	
Tier 7	X	X		

## Data Availability

The data and scripts that support the findings of this study are openly available on GitHub at https://github.com/isglobal-chagas/snake-epitopes (updated on 6 June 2022).

## Supplementary Materials

The following supporting information can be downloaded at: https://www.mdpi.com/article/10.3390/toxins14060418/s1. Supplementary Figure S1: Mapping of selected epitopes from PDB. Epitope location is highlighted in orange. PDB structures in parenthesis. Supplementary Figure S2: Mapping of selected epitopes from models. Epitope location is highlighted in orange. Reference sequence accession number and PDB structures in parenthesis. Supplementary Figure S3: Illustration of an example alignment depicting which residues were accounted for the entropy calculation. In blue, positions that are taken into consideration. In orange, not considered positions. Hyphens indicate gaps in the alignment, while X’s indicate unknown residues. Supplementary Figure S4: Illustration of epitope prediction. Sequences are broken into conserved fragments (asterisks indicate non-conserved residues), and each fragment is considered separately and analyzed 8-mer by 8-mer. Overlapping 8-mers are incorporated into the final epitopes if they fulfill any tier criteria. Struckthrough peptides represent discarded fragments due to their short length. In grey. 8-mers discarded for not fulfilling any tier criteria. or having a high blast identity. Bold peptides indicate the final predicted epitopes. Supplementary Table S1: Results of the PDB BLAST and protein modelling. Only clusters with a PDB hit identity between 90% and 50% were considered for modelling. N/A = not available. Supplementary Table S2: Selected epitopes list. Species covered are represented with their taxonomic identifier (taxid). Reference sequences indicate their NCBI id and description, and are classified as members of three-finger toxins (3FTX), phospholipase A2s (PLA2), snake venom metalloproteinases (SVMP), snake venom serine proteases (SVSP), Kunitz peptides (KUN), or other (OTHER). Species covered are indicated by their taxid for brevity. Epitopes that originate from several reference sequences are due to merging events. Supplementary Table S3: List of keywords used to identify the different protein families. Protein descriptions were compared with the keywords, and each protein was classified accordingly. Proteins that did not fit into any family were labeled as “OTHER”. Supplementary Table S4: Snake venom composition by species. Values adapted from the review by Tasoulis and Isbister [7]. Numbers indicate protein family fraction of total venom, distributed in Three-finger toxins (3FTX), Cysteine-rich secretory proteins (CRISP), disintegrins (DIS), Kunitz peptides (KUN), L-amino acid oxidases (LAAO), natriuretic peptides (NP), phospholipase A2s (PLA2), snake C-type lectins (SNACLEC), snake venom metalloproteinases (SVMP), snake venom serine proteases (SVSP), or other (OTHER). Species not present in this table were assigned values according to the mean proportions of their snake family (Viperidae and Elapidae). For *Atractaspis irregularis* (Lamprophiidae), the same proportions were assigned to each protein family. Supplementary File S1: Fasta file containing the polyepitope sequence and its corresponding DNA sequence, optimized for mice codon usage. Supplementary File S2: Multiple sequence alignments of clusters for the selected epitopes.
