# Supplementary material for: Computer-Aided Analysis of West Sub-Saharan Africa Snakes Venom towards the Design of Epitope-Based Poly-Specific Antivenoms
Source: Toxins (Basel). 2022 Jun 18;14(6):418. doi: 10.3390/toxins14060418 (PMC9229730; doi:10.3390/toxins14060418)
Supplement: Supplementary file 1 [file toxins-14-00418-s001.zip › Supplementary Figures.pdf]

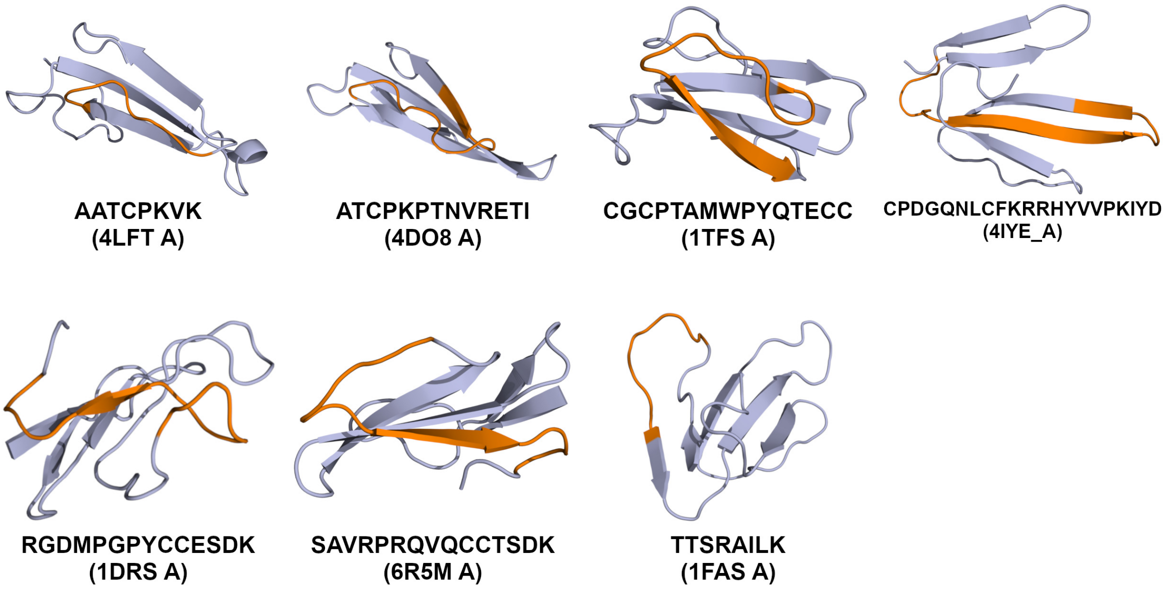

Figure S1. Mapping of selected epitopes from PDB. Epitope location is highlighted in orange. PDB structures in parenthesis.

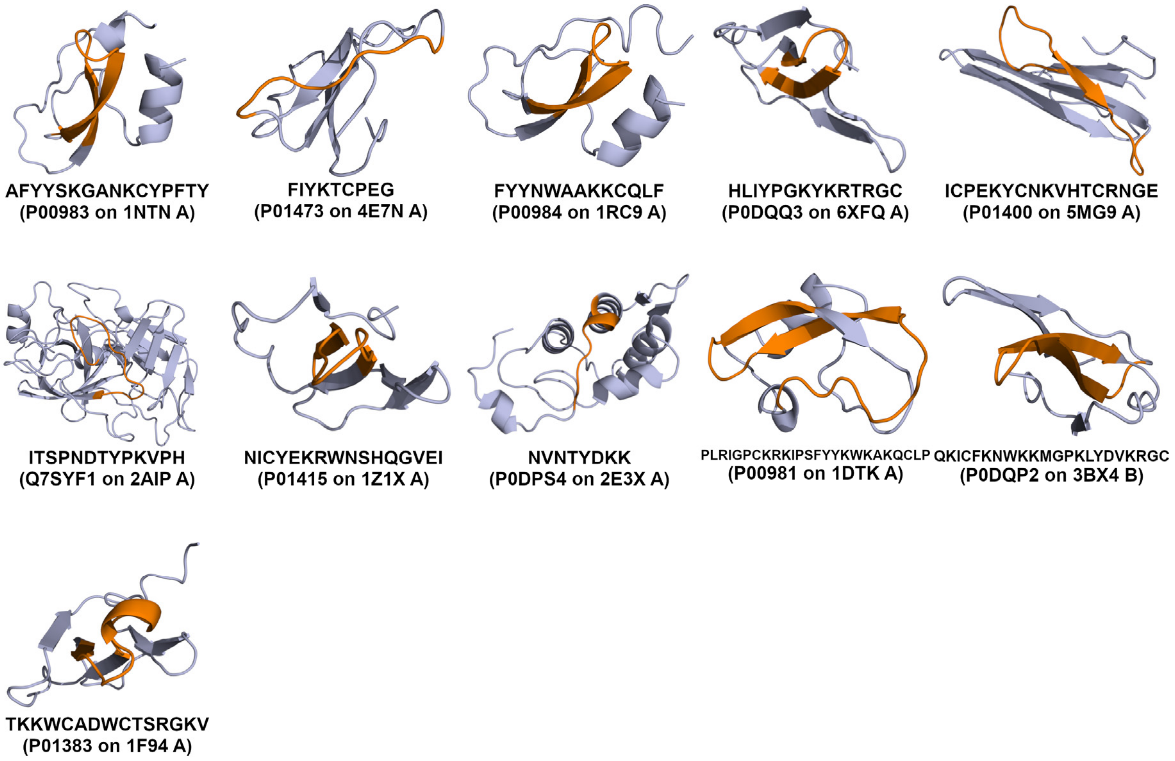

Figure S2. Mapping of selected epitopes from models. Epitope location is highlighted in orange. Reference sequence accession number and PDB structures in parenthesis.

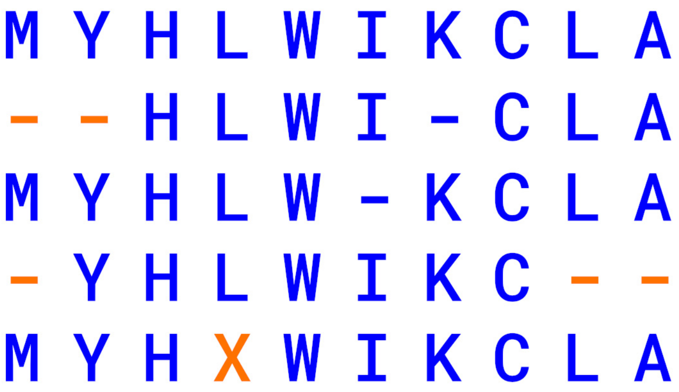

Figure S3. Illustration of an example alignment depicting which residues were accounted for the entropy calculation. In blue, positions that are taken into consideration. In orange, not considered positions.

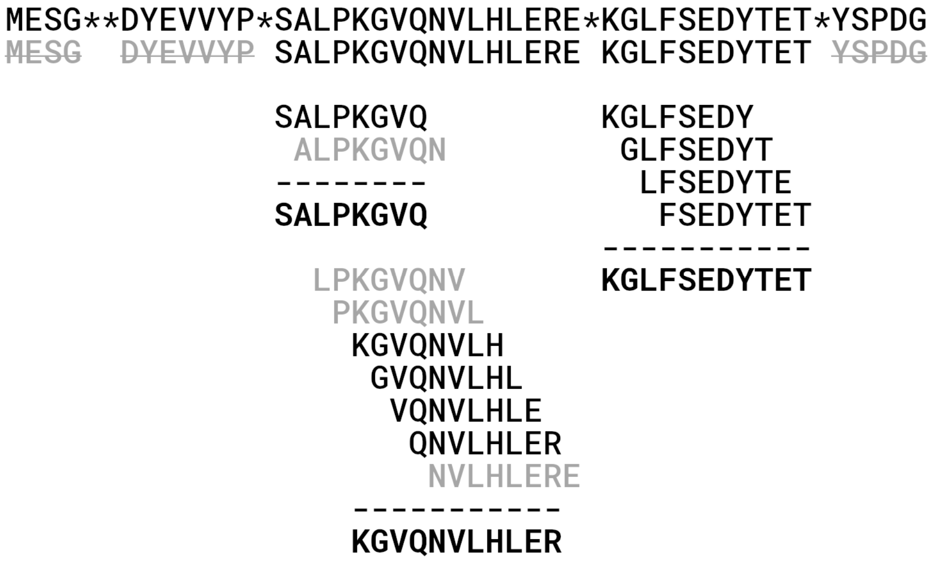

Figure S4. Illustration of epitope prediction. Sequences are broken into conserved fragments (asterisks indicate non-conserved residues), and each fragment is considered separately and analyzed 8-mer by 8-mer. Overlapping 8-mers are incorporated into the final epitopes if they fulfill any tier criteria. Struckthrough peptides represent discarded fragments due to their short length. In grey. 8-mers discarded for not fulfilling any tier criteria. or having a high blast identity. Bold peptides indicate the final predicted epitopes.
